# Long-term neurocognitive function after whole-brain radiotherapy in patients with melanoma brain metastases in the era of immunotherapy

**DOI:** 10.1007/s00066-022-01950-1

**Published:** 2022-05-11

**Authors:** Martin Salzmann, Klaus Hess, Kristin Lang, Alexander H. Enk, Berit Jordan, Jessica C. Hassel

**Affiliations:** 1 Department of Dermatology and National Center for Tumor Diseases, University Hospital Heidelberg, Im Neuenheimer Feld 460, 69120 Heidelberg, Germany; 2 Department of Neurology, University Hospital Heidelberg, Heidelberg, Germany; 3 Department of Radiation Oncology, University Hospital Heidelberg, Heidelberg, Germany; 4 Department of Neurology, Halle University Hospital, Halle (Saale), Germany

**Keywords:** Melanoma, Brain metastases, Cognitive functioning, Whole-brain radiotherapy, Neurocognition

## Abstract

**Background:**

Whole-brain radiotherapy (WBRT) used to be standard of care for patients suffering from melanoma brain metastases (MBM) and may still be applicable in selected cases. Deterioration of neurocognitive function (NCF) is commonly seen during and after WBRT. Knowledge on long-term effects in melanoma patients is limited due to short survival rates. With the introduction of immune checkpoint inhibitors, patients may experience ongoing disease control, emphasizing the need for paying more attention to potential long-term adverse effects.

**Methods:**

In this single-center study, we identified in a period of 11 years all long-term survivors of MBM who received WBRT at least 1 year prior to inclusion. NCF was assessed by Neuropsychological Assessment Battery (NAB) screening and detailed neurological exam; confounders were documented.

**Results:**

Eight patients (median age 55 years) could be identified with a median follow-up of 5.4 years after WBRT. Six patients reported no subjective neurological impairment. NAB screening revealed an average-range score in 5/8 patients. In 3/8 patients a NAB score below average was obtained, correlating with subjective memory deficits in 2 patients. In these patients, limited performance shown in modalities like memory function, attention, and spatial abilities may be considerably attributed to metastasis localization itself. Six out of 8 patients were able to return to their previous work.

**Conclusion:**

Five of 8 long-term survivors with MBM after WBRT experienced little to no restriction in everyday activities. In 3 out of 8 patients, cognitive decline was primarily explained by localization of the metastases in functionally relevant areas of the brain. The results of our small patient cohort do not support general avoidance of WBRT for treatment of brain metastases. However, long-term studies including pretreatment NCF tests are needed to fully analyze the long-term neurocognitive effects of WBRT

**Supplementary Information:**

The online version of this article (10.1007/s00066-022-01950-1) contains supplementary material, which is available to authorized users.

## Introduction

The treatment of metastasized melanoma (MM) has changed dramatically within the past decade, mainly due to the introduction of immune checkpoint inhibitors (ICI) and targeted treatment, leading to possible long-term remissions. This also includes the treatment of melanoma brain metastases (MBM) [1–3], the most aggressive subtype of metastases, with poor prognosis [4, 5]. About 50% of all MM patients develop MBM. For MBM, radiotherapy (RT) plays a major role in disease control [6, 7]. Historically, whole-brain radiotherapy (WBRT) was considered standard of care, especially for patients suffering from a high number of symptomatic MBM, and is still a potential treatment option [8, 9]. However, treatment is progressively switching to stereotactic radiosurgery (SRS) as the preferred treatment, especially in patients with a limited number of metastases [10, 11]. Adjuvant WBRT has been shown to be ineffective in patients with melanoma. Except for an increased local tumor control in patients with 1–3 MBM, the general outcome is not favorable [12]. Current European Society for Medical Oncology (ESMO) guidelines recommend avoiding WBRT in asymptomatic melanoma metastasis with regard to lack of efficacy and long-term toxicities [13].

Several prospective studies have compared the rate of neurocognitive function (NCF) decline of patients treated by SRS alone compared to patients receiving either SRS and WBRT in combination [11, 14] or SRS vs. WBRT [15]. Brown et al. [11] demonstrated superiority of SRS alone in terms of cognitive deterioration at 3 months from initiating RT; the differences at 12 months were still significant, yet less conclusive. However, all studies focused on short- to medium-term cognitive differences. As such, data on long-term functional outcomes with respect to NCF at more than 12 months are missing.

Through the introduction of novel treatment options like ICI, potential long-term survival and long-term toxicities such as neurocognitive outcome should be considered when discussing treatment options such as WBRT [16]. The aim of our study was to describe neurological function with focus on NCF in a series of MBM long-term survivors treated with WBRT.

## Patients and methods

### Patient population

This is a monocentric, cross-sectional analysis performed at the Section of Dermatooncology, Department of Dermatology and National Center for Tumor Diseases, and the Section of Neurology, University Hospital Heidelberg. We identified patients by data inquiry of all melanoma patients receiving WBRT between 2009 and 2020 with electronic records available at our institution. The neurocognitive assessments were done as a follow-up within routine clinical practice based on regular neurological consultation service. Patients were included retrospectively into this analysis. Patients were eligible with documented MM with MBM, currently in remission, and WBRT at least 1 year prior to inclusion. There was no restriction on the use of previous, concurrent, or subsequent systemic agents. Patients treated with WBRT both in a palliative and adjuvant setting were eligible. All patients were treated according to local standards at the time of therapy, which may no longer be standard of care. Patients were free of potentially confounding systemic inflammatory or metabolic disorders. Patients with a concomitant psychiatric disorder were excluded.

### Neurocognitive function testing

NCF was assessed with the screening part of the validated German version of the Neuropsychological Assessment Battery (NAB) [17], which includes detailed modules on attention, language, spatial abilities, memory, and executive functions, thus providing an estimate of the examinee’s functioning in the abovementioned domains. The tests were performed within clinical routine in the NAB screening module version [17], taking about 1 h and not including extensive module testing. All tests were performed by the same neuropsychologist (KH). Test scores were assessed as primary scores (T scores), which were referenced to the respective normative sample and standard scores for the respective patient group regarding age and sex. Each patient obtained a descriptive score of “average, low/high average, above/below average, and high above/high below average” according to the percentile reached for each test domain. Concurrent medication and secondary diagnoses were recorded, and a detailed neurological examination performed at the date of NCF testing. As the premorbid level of NCF was not available, the patients’ profession before developing melanoma was recorded as an indication of premorbid NCF. Subjective symptoms verbalized by the patients were assessed before NCF testing.

### Data collection

Additional collected data included core data (gender, age), data on the disease prior to initiation of therapy (date of first diagnosis, primary site, number of brain metastases, site of metastases), and on prior treatment lines. Date and details of WBRT were documented, as well as previous or subsequent RT, if performed, and complications of RT. The retrospective analysis of patient data was approved by the ethical committee of the Medical Faculty Heidelberg (S-454/2015).

## Results

A total of 184 patients treated at our institution received WBRT for MBM between 2009 and 2020; 11 patients (6%) were alive and in long-term observation and treatment at our institution. Of these, 2 developed progressive disease and were treated in regional hospitals, and 1 patient did not desire neurological consultation. Hence, we are able to report on 8 patients who received neurological assessment including NCF testing for either subjective deficits or differential diagnostic purposes. At the time of NCF assessment, patients did not receive any steroids nor any other drugs influencing neurocognitive function, except for two patients receiving anticonvulsants.

### Patient characteristics

Patient characteristics are shown in Table 1. Of 8 included patients, 7 were male, with a median age of 55 years (range 45–64). The median duration of follow-up was 5.4 years after RT (range 1.0–12.3).

**Table 1:** Patient characteristics

ID	Sex	Age (years)	Indication for WBRT	Number of brain metastases prior to RT	Largest diameter MBM prior to RT	Localization of largest MBM	Date of WBRT	Cumulative WBRT dose	RT fractions	Concurrent treatment	S100 serum levels, xULN (before RT)	LDH serum levels, xULN (before RT)	Systemic treatment before RT	Other systemic treatment after RT	Duration of follow-up (years)
1	Male	64	Adjuvant	0 (1 resected)	2.5 cm	Temporo-parietal cortical (left)	03/2008	37.5 Gy (+ 9 Gy to resection area)	15 (+ 4)	Temozolomide	Not available	Not available	Interferon‑α, dacarbazine	None	12.3
2	Female	56	Palliative	5 (1 resected)	0.4 cm	Parieto-occipital cortical (left, resected)	11/2009^a^	30 Gy	10	Dacarbazine	Not available	Not available	None	Carboplatin/paclitaxel, ipilimumab	10.4
3	Male	63	Palliative	4	1.5 cm	Temporobasal (right)	11/2013	30 Gy	10	Ipilimumab	Not available	0.77	None	None	6.8
4	Male	48	Palliative	3 (1 resected)	0.4 cm	Occipital cortical (left, resected)	07/2014^a^	30 Gy	10	Ipilimumab	0.51	0.87	Interferon‑α	Dabrafenib/trametinib, pembrolizumab	5.8
5	Male	60	Adjuvant	0 (1 resected)	3.3 cm	Occipital cortical (right)	05/2015	30 Gy	10	Ipilimumab	5.50	1.16	None	None	5.0
6	Male	51	Palliative	> 10	3.5 cm	Frontal cortical (left), bifrontal, biparietal	09/2015	30 Gy	10	Ipilimumab + nivolumab	1.86	1.09	None	None	4.6
7	Male	45	Palliative	4	2.4 cm	Fourth ventricle (left), left temporal	12/2015^a^	30 Gy (+ 9 Gy to vermis)	10 (+ 3)	Ipilimumab+ nivolumab	Not available	0.50	None	None	4.4
8	Male	54	Palliative	15	0.5 cm	Parietal cortical right	04/2019	30 Gy	10	Ipilimumab	1.10	0.72	Dabrafenib/trametini + PD‑1 inhibitor (clinical trial)	Vemurafenib/cobimetinib	1.0

*Gy* Gray, *LDH* Lactate dehydrogenase, *MBM* Melanoma brain metastases, *MM* Metastasized melanoma, *NCF* Neurocognitive function, *RT* Radiotherapy, *SRS* Stereotactic radiosurgery, *ULN* Upper limit of normal, *WBRT* Whole-brain radiotherapy

^a^Patients 4 and 7 received subsequent SRS to singular progressive metastases (patient 4: occipital left, March 2015, 30 Gy; patient 7: frontal right, January 2019, 20 Gy); patient 2 had received SRS of a parietooccipital left metastasis prior to WBRT in November 2008 (20 Gy)

### Brain metastases and radiotherapy

Of 8 patients, 6 were treated with palliative intention and two in an adjuvant intention after resection of a singular metastasis. The patients treated in palliative intention had a medium of 4.5 metastases (range 3–15). Seven patients were treated with WBRT to a cumulative radiation dose of 30 Gy applied in 10 fractions. One patient received a boost of 9 Gy to a symptomatic metastasis of the vermis. One patient treated in 2008 received a regimen of 37.5 Gy applied in 15 doses, with an additional boost of 9 Gy in 4 doses to the resected area. Patients 1, 3, and 6 required dexamethasone treatment (maximum doses of 4, 8, and 16 mg per day, respectively) for neurological symptom relief before RT or surgical resection. Patients 2 and 4 had one metastasis resected but received WBRT due to further MBM as shown in Table 1.

### Systemic treatments and treatment outcome

**Fig. 1:**
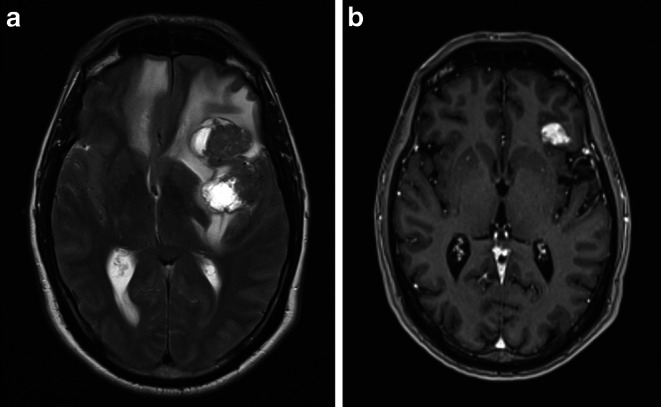
**a** Brain MRI (fluid-attenuated inversion recovery, FLAIR) of patient 6 in August 2015 before the onset of treatment. Several large brain metastases with perifocal FLAIR hyperintensities in the frontal and parietal lobe, the largest left frontal metastasis measuring 22 × 34 mm. Midline shift to the right by 12 mm and compression of the left lateral ventricle and the third ventricle. The patient was admitted to neurological inpatient care due to symptoms of cerebral compression, which were compensated by dexamethasone treatment. **b** Brain MRI (T1 with contrast enhancement) in August 2020 at the time of neurocognitive function testing. Residual, stable brain metastases after radiotherapy and checkpoint inhibitor treatment with ipilimumab and nivolumab. At this point, the patient had an overall average neurocognitive function, allowing a return to his job as a teacher

The two patients treated before 2013 received chemotherapy-based systemic treatment regimens (temozolomide, dacarbazine), with one of them receiving subsequent ipilimumab leading to complete remission. All patients treated in 2013 or later received an ipilimumab-based systemic checkpoint inhibitor treatment. In patient 4, an ongoing remission was achieved with the addition of pembrolizumab monotherapy; patient 8 was in remission at the time of data collection during systemic treatment with dabrafenib and trametinib, but progressed shortly after. All other patients were still in an ongoing remission at the time of final follow-up in June 2021. Patient 6, who had the highest burden of intracranial disease among our patients (Fig. 1), is still continuing treatment with nivolumab 240 mg every 4 weeks.

### Neurological findings and neurocognitive outcome

A summary of neurological and neurocognitive outcomes is given in Table 2. Neurological function was normal in 5 of 7 patients, including 3 patients with mild central paresis signs not leading to disability in everyday functioning. Patient 4 suffered from quadrant anopsia related to occipital metastasis manifestation. Patients 4 and 8 developed symptomatic epilepsy based on the metastatic brain lesions. Permanent seizure control with anticonvulsants (levetiracetam 2500 mg/d and 2000 mg/d respectively) was achieved, yet the ongoing treatment might be a confounder to neurocognitive function.

**Table 2:** Results of neurological examination and neurocognitive assessment

ID	Subjective impairment of NCF	Neurological examination and additional remarks	Neurocognitive assessment (Neuropsychological Assessment Battery)						Premorbid level
			Overall	Attention	Language	Memory	Spatial	Executive	
1	Memory deficits, susceptibility to errors	Normal	*Below average*	Low average	Average	Below average	Low average	Low average	University degree/business engineer^a^
2	None	Mild central left leg paresis after intracranial hemorrhage	*Below average*	Below average	Average	Average	Below average	Below average	Qualified job/nurse^a^
3	None	Normal	Low average	Below average	Average	Low average	Average	Average	University degree/business economist^a^
4	Memory deficits, amnestic aphasia, mood swings	Slight quadrant anopsia to lower right, mild sensory polyneuropathy of the legs; symptomatic epilepsy	*Below average*	Far below average	Low average	Far below average	Low average	Low average	Qualified job/scaffolder + baker
5	None	Not done	Above average	High average	High average	Average	Far above average	High average	University degree/architect^a^
6	None	Slight paresis of the right leg	Low average	Average	Average	Average	Below average	Average	University degree/teacher^a^
7	None	Normal	Average	Low average	Average	Average	Average	Average	University degree/interior designer^a^
8	None	Sensory more than motor polyneuropathy predominating in the legs (confounder: autoimmune polyneuropathy during PD1-treatment), symptomatic epilepsy	Average	Low average	Average	High average	High average	Average	University degree/chemical engineer

*NCF* Neurocognitive function

^a^Patient returned to their job

In NAB screening, 5/8 patients achieved an average score or higher (Table 2). In 3 patients (ID 1, 2, 4), an overall score below average was reached; in correlation, 2 of them (patients 1, 4) also reported subjective cognitive impairment, especially deficits in memory (Table 2). All other patients felt no subjective impairment in neurocognition in everyday life.

All patients presented with a high premorbid cognitive level, as assessed by their profession: 6 patients held a university degree, while 2 worked in qualified jobs as a nurse and scaffolder. Six of these patients were able to continue their jobs, patient 4 is on disability pension, and patient 8 could not return to work due to progression of the underlying disease. This patient previously developed autoimmune polyneuropathy after treatment with PD1-inhibitors, as a potential confounder in neurological examination.

Detailed results of the NAB screening are described in the supplements (Supplementary Table 1).

## Discussion

In this study, we present a group of 8 patients with long-term survival after WBRT for MBM. When discussing the results, it should be noted that the patient sample is small, which represents a limitation of our study. Therefore, any conclusions based on these data need to be taken with care.

It is remarkable that in 6/8 patients, neurological outcome and especially neurocognitive function after WBRT allowed patients return to their previous work. Further, most patients reported no subjective impairment in neurocognitive function.

Simultaneously, we also identified 3/8 patients (ID 1, 2, 4) with reduced neurocognitive function in NAB screening after WBRT (Table 2; Supplementary Table 1). Subjective memory deficits in patients 1 and 4 are mirrored in corresponding below average scores in the memory module (Table 2). Both patients noted cognitive deterioration soon after WBRT, showing an impact on their everyday life. Thus, a relevant proportion of patients (38% of our cohort) still had a low-average to below-average neurocognitive function, and 2 patients (25%) had relevant impairment of their everyday life. It has to be noted that premorbid NCF was not available for the analysis. As such, other causes beside WBRT have to be considered and will be discussed below.

In general, up to two thirds of patients with brain metastases experience neurocognitive impairment within 2–6 months after WBRT, including concentration deficits but also decreased short- and long-term memory [11, 14, 18, 19].

However, in our patients, the cause of cognitive deterioration should not only be seen in potential radiogenic toxicity. As such, parietal lesion localization itself may certainly at least partially explain deficits in spatial skills (patient 1, 2; e.g., identifying and self-constructing different patterns in NCF testing), reduced memory performance of patterns (patient 1), and also reduced attention (patients 2, 4: marking “X” and “numbers” in 16 and 8 rows, respectively, in the NCF test battery). In addition, in patient 4, a partial visual field defect due to a metastatic lesion at the visual cortex might contribute to reduced visual ability to identify objects as well as levetiracetam intake reducing working memory and attention in some patients. Further, in comparison to the other patients, in patient 1, the WBRT treatment dose was increased and surgical resection of parietal metastasis was performed (Table 1).

Decreased NCF may also be based on concurrent systemic anticancer treatments. As shown in Table 1, 7 of 8 patients (88%) received ICI, for which neurological adverse events are rare, but may be severe [20]. An influence of ICI on neurocognitive function is rare [21] and to the best of our knowledge, has not been evaluated systematically so far. In our cohort, only 1 patient (patient 8) suffered from ICI-induced sensorimotor neuropathy as an autoimmune related adverse event, which required temporary systemic steroids and had completely resolved 6 months before NCF testing. Further, an influence of chemotherapy on NCF might be discussed as well, but may be especially relevant in elderly patients [22]. It is rather unlikely that concurrent chemotherapy with temozolomide and dacarbazine (in patients 1 and 2, respectively) can explain a cognitive impairment but may of course be a contributing factor.

Temozolomide is even expected to prevent neurocognitive decline in patients with primary or metastatic CNS tumors [23–25]. However, cognitive changes associated with cancer treatment may be diffuse and the topic of chemobrain is broad [21, 26].

Neurotoxic deficits usually involve the domains of attention and concentration, verbal and visual memory, and processing speed. Two of our patients had received targeted treatment with BRAF and MEK inhibitors in the past, which may rarely cause central neurotoxicity [21] as well as paclitaxel in very rare cases.

Certainly we acknowledge that 75% of our patients held a university degree and would be expected to have a higher-than-average premorbid neurocognitive function. This might be a clinical hint toward potential deterioration after WBRT, as frequently seen. Also, it has to be noted that our cohort was highly selected and included potentially very fit patients; similar to toxicity seen in chemotherapy, more frail patients may suffer many more short- and long-term side effects from WBRT. Without any premorbid NAB status available, we cannot prove a deterioration of NCF over the course of treatment without a full return to normal. In patient 6, high intracranial tumor load as well as bifrontal and biparietal localization of metastases may explain the worse cognitive performance than expected with regard to profession as a teacher.

When deciding on an optimal treatment for each patient, potential deterioration of NCF always has to be considered. Short-term deterioration of NCF due to WBRT is well recognized and has been shown in randomized controlled trials [11, 14]. However, long-term effects in melanoma patients are widely unknown, as previously highly unlikely without an effective anticancer systemic treatment. Thus, factors predictive of long-term neurocognitive toxicity of WBRT are poorly studied, especially in melanoma [21]. Several studies have evaluated potentially beneficial additions to WBRT to preserve neurocognition, mainly including the use of systemic memantine [27]. A hippocampal-sparing technique in WBRT to avoid cognitive decline, especially in memory, should be preferred and is recommended [28–30].

Patients studied in our case series mostly show a favorable outcome with good everyday functioning and return to activities. This is in line with Jiang et al. reporting on beneficial outcomes in MBM patients after WBRT, showing only limited neurocognitive side effects. However, neurocognitive function was not measured objectively [31]. Favorable outcomes of children treated with cerebral RT have been described as well [32].

These findings may encourage consideration of WBRT as a potential treatment option in selected cases, as the goal in melanoma treatment is progressively switched to long-term tumor control, even in patients with MBM.

Meanwhile, there is also a large body of evidence suggesting progressive use of SRS as the primary treatment option in combination with systemic treatment [33]. This does not only include a limited number of 1–3 brain metastases; SRS can currently be used safely in up to 15 brain metastases [34–36]. While radionecrosis may be a factor to consider in SRS [37], the main advantage of SRS versus WBRT is reduced neurotoxicity, which has been demonstrated in several studies, especially confirming short-term neurocognitive decline induced by WBRT [35]. Therefore, studies on long-term neurocognitive decline after WBRT are needed especially in tumors aiming for long-term tumor control.

Especially in patients with brain metastasis in whom local therapy has failed, or with neurological symptoms requiring steroids or leptomeningeal disease, an infrequent response to ipilimumab/nivolumab has been shown [38]. This population can be treated by WBRT, even in the case of leptomeningeal disease or very extensive involvement.

We are aware of limitations of this analysis, including the very small number of patients, the partial bias due to localization of brain metastasis in neuropsychologically relevant areas, and the lack of premorbid NCF, precluding a general conclusion on long-term effects of WBRT for MBM. The strength of our study was the differentiated neurocognitive test battery applied to all long-term survivors, allowing a validated statement on the neurocognitive function of these patients.

## Conclusion

Generally impaired NCF in long-term survivors with MBM after WBRT could not be confirmed in our study. In light of the favorable neurocognitive function outcome in our small long-term survivor cohort, WBRT in MBM still seems to be a valid treatment option. Therefore, WBRT should still be considered in the treatment armamentarium for melanoma patients with symptomatic brain metastases. However, long-term studies including pretreatment NCF tests are needed to fully analyze the long-term neurocognitive effects of WBRT.

## Supplementary Information

Supplementary Table 1: T scores (T) and percentiles (P) of all patients for the performed screening modules.

## Abbreviations

ICI: Immune checkpoint inhibitor
MBM: Melanoma brain metastases
MM: Metastasized melanoma
NAB: Neuropsychological Assessment Battery
NCF: Neurocognitive function
RT: Radiotherapy
SRS: Stereotactic radiosurgery
WBRT: Whole-brain radiotherapy
