# Supplementary material for: Long-term neurocognitive function after whole-brain radiotherapy in patients with melanoma brain metastases in the era of immunotherapy
Source: Strahlenther Onkol. 2022 May 11;198(10):884–91. doi: 10.1007/s00066-022-01950-1 (PMC9515012; doi:10.1007/s00066-022-01950-1)
Supplement: Supplementary file 1 — Supplementary Table 1: T scores (T) and percentiles (P) of all patients for the performed screening modules. [file 66_2022_1950_MOESM1_ESM.docx]

Supplementary Table 1: T-Scores (T) and percentiles (P) of all patients for the performed screening modules.

| Patient | **NAB screening test module** | | | | | | | | | | |
| --- | --- | --- | --- | --- | --- | --- | --- | --- | --- | --- | --- |
|  | **Attention: numbers and digits** | | | **Language** | | **Memory** | | **Executive functions** | | **Spatial** | |
|  | Marking X, part A, form 1 | Counting X, part B, form 1 | Repeating numbers  forwards/  backwards Part B, form 1 | Naming objects on pictures, form 1 | Speech production, form 1 | Shape learning  (immediate/ delayed), form 1 | Story learning  (immediate/delayed), form 1 | Word fluency, form 1 | Labyrinth, form 1 | Identifying a complex figure, form 1 | Recreating a figure pattern, form 1 |
| Patient 1 | T: 30  P: 2 | T: 33  P: 4 | T: 57 / 60  P: 76 / 84 | T: 49  P: 46 | T: 45  P: 31 | T: 35 / 39  P: 7 / 14 | T: 43 / 44  P: 24/27 | T: 50  P: 50 | T: 38  P: 12 | T: 40  P: 16 | T: 45  P: 31 |
| Patient 2 | T: 37  P: 10 | T: 44  P: 27 | T: 41 / 42  P: 18 / 21 | T: 48  P: 42 | T: 48  P: 42 | T: 51 / 37  P: 54 / 10 | T: 57 / 59  P: 76 / 82 | T: 38  P: 12 | T: 40  P: 16 | T: 32  P: 4 | T: 38  P: 12 |
| Patient 3 | T: 42  P: 21 | T: 36  P: 8 | T: 32 / 38  P: 4 / 12 | T: 51  P: 54 | T: 51  P: 54 | T: 52 / 54  P: 58 / 66 | T: 34 / 40  P: 6 / 16 | T: 35  P: 7 | T: 58  P: 79 | T: 51  P: 54 | T: 40  P: 16 |
| Patient 4 | T: 31  P: 3 | T: 24  P: 1 | T: 45 / 47  P: 31 / 38 | T: 38  P: 12 | T: 50  P: 50 | T: 42 / 36  P: 21 / 8 | T: 27 / 28  P: 1 / 1 | T: 45  P: 31 | T: 41  P: 18 | T: 39  P: 14 | T: 47  P: 38 |
| Patient 5 | T: 46  P: 34 | T: 52  P: 58 | T: 60 / 70  P: 84 / 98 | T: 60  P: 84 | T: 58  P: 79 | T: 59 / 46  P: 82 / 34 | T: 52 / 53  P: 58 / 62 | T: 58  P: 79 | T: 55  P: 69 | T: 63  P: 90 | T: 69  P: 97 |
| Patient 6 | T: 44  P: 27 | T: 42  P: 21 | T: 60 / 54  P: 84 / 66 | T: 48  P: 42 | T: 51  P: 54 | T: 51 / 46  P: 54 / 43 | T: 44 / 43  P: 27 / 24 | T: 56  P: 73 | T: 40  P: 16 | T: 40  P: 16 | T: 31  P: 3 |
| Patient 7 | T: 43  P: 24 | T: 39  P: 14 | T: 50 / 41  P: 50 / 18 | T: 60  P: 84 | T: 48  P: 42 | T: 58 / 52  P: 79 / 58 | T: 51 / 49  P: 54 / 46 | T: 47  P: 38 | T: 57  P: 76 | T: 50  P: 50 | T: 53  P: 62 |
| Patient 8 | T: 34  P: 4 | T: 42  P: 21 | T: 46 / 48  P: 31 / 42 | T: 60  P: 84 | T: 48  P: 42 | T: 68 / 61  P: 96 / 86 | T: 57 / 57  P: 76 / 76 | T: 46  P: 31 | T: 46  P: 31 | T: 64  P: 90 | T: 60  P: 84 |
